# Methemoglobinemia due to use of poppers: a case report

**DOI:** 10.1186/s13256-022-03475-8

**Published:** 2022-06-21

**Authors:** Emmanuelle Sonck, Eric Bourmanne, Julien Bruteyn, Waheed Dolip

**Affiliations:** 1 Centre Hospitalier Epicura – site d’Ath, Rue Maria Thomée 1, 7800 Ath, Belgium; 2 Service des Urgences, Centre Hospitalier Epicura – site d’Ath, 1 rue maria thomée, 7800 Ath, Belgium

**Keywords:** Methemoglobinemia, Intoxication, Nitrites, Cyanosis, Methylene blue

## Abstract

**Background:**

Methemoglobinemia is an excess of oxidized hemoglobin in the blood, affecting oxygen transportation. It is characterized by central cyanosis that does not respond to oxygen therapy. Prognosis is excellent when treated adequately and rapidly. We present a case report of a 38-year-old Caucasian man suffering from methemoglobinemia due to the use of poppers.

**Case presentation:**

A 38-year-old Caucasian man known as a smoker and addicted to cocaine was admitted to the emergency department with dyspnea, agitation, and central cyanosis that started approximately 3 hours before admission. The persistent hypoxia despite high-flow oxygen therapy and a history of poppers use helped to reveal a condition known as methemoglobinemia.

**Conclusions:**

Our case highlighted a typical clinical presentation of methemoglobinemia. This possible life-threatening condition can occur after ingestion or inhalation of poppers, commonly sold in sex shops for recreational purposes. This can be easily confirmed by the methemoglobin level of the blood gases, provided the emergency physician considers this diagnosis. Rapid treatment with intravenous methylene blue is effective and leads to a favorable prognosis.

## Background

Methemoglobinemia is a condition in which iron in hemoglobin is oxidized from the ferrous state to the ferric state, making it unfit to carry oxygen throughout the body [1–5]. It can be caused by various factors, either congenital or by intoxication. Among the nonorganic toxicants that can cause methemoglobinemia are poppers.

We decided to publish this case report because of the rarity of this pathology and given the incidence of poppers use, especially among the male homosexual population [3, 5, 6]. It is a medical emergency whose clinical presentation is characteristic and will allow a rapid diagnosis, with appropriate treatment leading to a favorable prognosis.

## Case presentation

A 38-year-old Caucasian man known as a smoker and addicted to cocaine was admitted to the emergency department with dyspnea, agitation, and cyanosis approximately 3 hours before admission. Upon interrogation, the patient did not reveal any known history of medical, surgical, or psychiatric disease.

On admission, blood pressure was 150/80 mmHg, heart rate 118 beats per minute, respiratory rate 32 breaths per minute, oxygen saturation at 72% on room air, and central temperature 36.9 °C. Physical examination showed normal lung and heart sound, and abdominal region did not show any guarding or rebound, but there was a central cyanosis visible on his hands, mouth, and lips with a slate-gray coloration of the skin. The neurological examination was normal.

He was put on 15 L of oxygen via a nonrebreathing mask before analysis of arterial blood gas. Complete blood count (CBC) was normal. The blood gas analysis disclosed a pH 7.45 (7.35–7.45), pCO_2_ 37.0 mmHg (35–45 mmHg), pO_2_ 341.0 mmHg (75–90 mmHg), HCO_3_ 25.0 mmol/L (22.0–29.0 mmol/L), Base excess (BE) 1.9 mmol/L (−2 to 2 mmol/L), SaO_2_ 97.4% (95–99%), and lactate 2.5 mmol/L (0.50–2.00 mmol/L). A chest x-ray, an electrocardiogram (EKG), and complete blood work were ordered. The x-ray showed normal lung tissue without any signs of pneumothorax, infection, or an enlarged heart. The EKG revealed a sinus tachycardia, and the blood test was completely normal. Urine drug analysis did not reveal any drugs. Despite a pO_2_ level at 341 mmHg, the clinical signs of hypoxia did not improve with oxygen therapy. All things considered, and knowing that the patient had a history of poppers use from a past visit to the emergency department, a dosage of methemoglobin was ordered, and it turned out to be at 35.2%. Later, the patient did confirm the use of a 15-mL popper prior to anal sexual intercourse, after which all his symptoms began.

A diagnosis of methemoglobinemia was proposed, and therefore a treatment with intravenous administration of methylene blue at a dose of 1 mg/kg was given over 15 minutes. The symptoms resolved rapidly owing to normalization of the methemoglobin level. The patient was later discharged home on the same day after a complete resolution of his symptoms.

## Discussion

Cyanosis of central origin can have different causes, as summarized in the table below. In our clinical case, the cause was methemoglobinemia of toxic origin.

**Table:** 

Causes of central cyanosis [1, 2, 7]	
Cardiac	∙ Hemodynamic pulmonary edema
	∙ Cardiac malformations with arteriovenous shunt
Pulmonary	∙ Pneumonia
	∙ Respiratory insufficiency
	∙ Pulmonary embolism
*Methemoglobinemia*	
Congenital	∙ NADH diaphorase enzyme deficiency, transmitted in an autosomal recessive mode, in these subjects the methemoglobin level varies between 20% and 45%
	∙ Structural abnormalities of hemoglobin that will favor the ferric form of heme
Nonorganic causes	∙ Poppers, nitrates (plant fertilizers, Ajax), gunpowder, chlorates
Organic causes	∙ Anesthetics (benzocaine, lidocaine, prilocaine) quinines and derivatives, metoclopramide, sulfonamides and sulfone (dapsone), phenazopyridine, phenylacetamide and derivatives, aminobenzene (aniline, naphthalene), nitrobenzene and derivatives, nitrotoluene.

Methemoglobin is an oxidized form of hemoglobin where ferrous iron Fe^2+^ is converted to ferric iron Fe^3+^, making it unsuitable for oxygen (O_2_) transport [2–5, 8]. During normal metabolism, a slight proportion of methemoglobin exists in the blood, for example, 1% in adults, 1.5% in newborns, and 2% in premature babies. It is permanently reduced by various enzymatic mechanisms.

The main mechanism is the nicotinamide adenine dinucleotide (NADH)-dependent methemoglobin reductase I or diaphorase, which captures electrons from NADH to reduce methemoglobin to hemoglobin. NADH is produced by the main pathway of glucose degradation. This system ensures the reduction of 95% of the physiological circulating methemoglobin, coming from methemoglobinizing products of the food of nitrifying bacteria [2–5].

**Table:** 

Symptomatology according to methemoglobin level [8, 1, 2, 9]	
15–30%	∙ Cyanosis, asthenia, dizziness, headache
30–50%	∙ Dyspnea, tachypnea, syncope
50–70%	∙ Obnubilation, coma, convulsions, circulatory failure, rhythm disorders
Over 70%	∙ Risk of death

The accessory mechanism uses the nicotinamide adenine dinucleotide phosphate (NADPH) methemoglobin reductase. The NADPH is supplied by the accessory pathway of glucose degradation, of which glucose-6-phosphate dehydrogenase (G6PD) is a key enzyme [2–5].

**Fig. 1:**
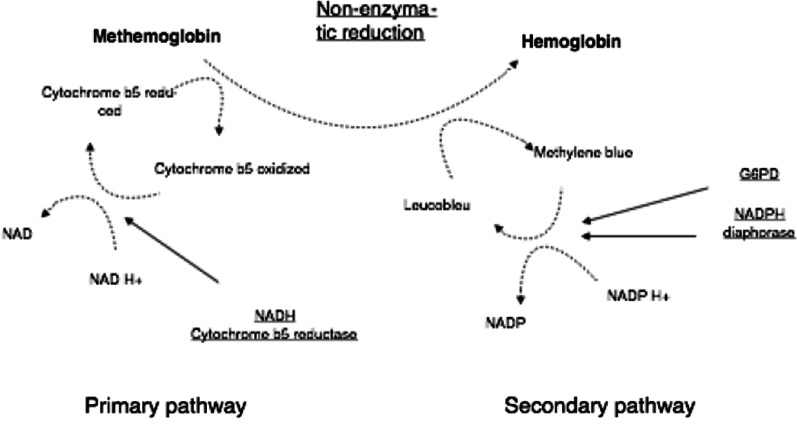
Pathways of methemoglobin reduction [10]. *NAD* nicotinamide adenine dinucleotide, *NADP* nicotinamide adenine dinucleotide phosphate, *G6PD* glucose-6-phosphate dehydrogenase

NADPH will allow the reduction of methylene blue to leucoblue, which will, in turn, reduce methemoglobin to hemoglobin. In the physiological state, this pathway is little used because it requires an electron carrier that is not available in the body (vitamin C or methylene blue) [2–5]. The treatment with methylene blue will allow maximum use of this pathway (Fig. 1) [1, 2, 4].

A French study in 2018 showed that more than 50% of cases of methemoglobinemia with values above 25% in the blood are linked to the use of poppers [8]. In the adult population as in teenagers, poppers are the second recreational drug taken after cannabis, confirming that it is not restricted to the homosexual community [11].

Poppers are an aliphatic nitrite (amyl, butyl, isobutyl, propyl), vasodilator, and oxidant [3, 5]. They come in the form of a volatile liquid to be inhaled in 10–15 mL vials and are sometimes used as a spray. The pop is the characteristic noise made by the vial being uncorked that gave them their name. Their euphoric and muscle-relaxing properties make it a very popular drug, particularly among the male homosexual population. The half-life is 14 minutes and has very short onset of action. It has hepatic metabolization. Vasodilatation will lead to some side effects, including facial flush, hypotension, tachycardia, headache, and nausea [3, 5].

The way that poppers work is through the rapid decomposition of the aliphatic nitrate, which is most commonly amyl nitrite to nitrous oxide. Another less common aliphatic nitrite is isopropyl nitrite, and it has a slower decomposition. The exact content of the amyl nitrate in poppers is not known. The nitrous oxide produced is responsible for the smooth-muscle relaxation among users. However, nitrous oxide is also responsible for the oxidation of ferrous iron to ferric iron that will transform hemoglobin into methemoglobin, making it unfit for oxygen transport [2–5, 8].

The cyanosis caused by methemoglobinemia is explained by a decrease in O_2_ bound to hemoglobin, causing a leftward shift in the Hb dissociation curve owing to an increased affinity of the unoxidized heme to O_2_, thus resulting in a decrease in oxygen supply to the periphery [8].

Generalized cyanosis is characteristic; in the absence of respiratory pathology, it suggests methemoglobinemia [4, 7–9, 12]. The chocolate color of the blood sample due to ferrous iron is also characteristic of methemoglobinemia [7–9, 12].

Methemoglobinemia will therefore cause respiratory distress that is unresponsive to O_2_ administration at normal oxygen pressure. Usually, arterial blood gas discloses a normal PaO_2_ [4, 5, 9, 13].

Hypoxia is a common clinical sign, even though arterial blood gas (ABG) discloses normal PO_2_. This physiologically appropriate PaO_2_ on ABG with low pulse oximeter saturation is called saturation gap.

In this case, the patient fulfilled all the clinical signs, and the other clinical investigations such as the blood test, chest x-ray, and EKG being normal, together with his history of poppers use, made this diagnosis very plausible. The treatment of methemoglobinemia consists of the intravenous administration of methylene blue that acts as a cofactor in the intra-erythrocyte reduction of methemoglobin in the presence of NADPH in non-G6PD-deficient subjects [1, 2, 4, 8].

When methemoglobinemia is symptomatic or if the level is over 30%, a methylene blue intravenous perfusion is given at the dose of 1–2 mg/kg over 15 minutes, possibly repeated after 1 hour if clinical signs persist, with a maximal dose of 7 mg/kg. More than this dose of methylene blue may also cause methemoglobinemia hemolysis. When methylene blue is ineffective, such as in cases of G6PD deficiency, a complete blood exchange can be performed [1, 2, 4, 8].

## Conclusion

Our case highlighted the typical clinical presentation of methemoglobinemia. This possibly life-threatening condition can occur after ingestion or inhalation of poppers, commonly sold in sex shops for recreational purposes. When a patient presents with central slate-gray cyanosis, lack of response to oxygen administration, chocolate-colored blood, and arterial blood gas showing unexplained hypoxia with normal PaO_2_, methemoglobinemia must be suspected.

This will be confirmed by the dosage of methemoglobin level in the blood. A careful history will usually identify the toxic agent. Rapid treatment with intravenous methylene blue is effective and leads to a favorable prognosis. It is therefore crucial for every emergency physician to be able to identify this situation in order to manage it adequately.

## Data Availability

EpiCura Ath hospital, Belgium, data available on request.
